# Sub-toxic cisplatin concentrations induce extensive chromosomal, nuclear and nucleolar abnormalities associated with high malignancy before acquired resistance develops: Implications for clinical caution

**DOI:** 10.1371/journal.pone.0311976

**Published:** 2024-12-26

**Authors:** John G. Delinassios, Robert M. Hoffman, George Koumakis, Dimitrios Palitskaris, Kyriaki-Nefelli Poulatsidou, George J. Delinasios

**Affiliations:** 1 International Institute of Anticancer Research, Kapandriti, Attica, Greece; 2 Department of Surgery, University of California, La Jolla, California, United States of America; 3 AntiCancer Inc., San Diego, San Diego, California, United States of America; Columbia University Irving Medical Center, UNITED STATES OF AMERICA

## Abstract

**Aim:**

This study investigates the impact of sub-toxic cisplatin levels on nuclear and nucleolar abnormalities and chromosome instability in HeLa cells since our current knowledge of cisplatin effects on these parameters is based on studies with high concentrations of cisplatin.

**Materials and methods:**

HeLa cells were exposed to gradually increasing sub-toxic doses of cisplatin (0.01 to 0.2 μg/ml). Cells treated with 0.1 and 0.2 μg/ml, termed HeLaC0.1 and HeLaC0.2, were not cisplatin-resistant, only exhibiting a slightly reduced viability, and were termed “cisplatin-sensitized cells.” Giemsa and silver staining were used to detect nuclear and nucleolar abnormalities and chromosomal alterations.

**Results:**

Notable abnormalities were observed in HeLaC0.1 and HeLaC0.2 cells after treatment with sub-toxic concentrations of cisplatin: nuclei showed abnormal shapes, blebs, micronuclei, fragmentation, pulverization, and multinucleation; nucleoli exhibited irregular shapes and increased numbers; anaphase cells showed more nucleolar organizing regions. Abnormal chromosome segregation, heightened aneuploidy (81–140 chromosomes), polyploidy, double minutes, dicentrics, chromatid exchanges, chromatid separations, pulverization, and chromosome markers were prominently noted. These abnormalities were intensified in cells pre-sensitized to 0.02 or 0.08 μg/ml cisplatin for seven days, then exposed to 0.03 or 0.1 μg/ml cisplatin for 24 hours, and finally cultured in cisplatin-free medium for 24 hours before chromosome analysis.

**Conclusion:**

HeLa cells subjected to increasing concentrations of sub-toxic cisplatin exhibited large-scale, multiple-type abnormalities in nuclei, nucleoli, chromosomes, and chromosomal numbers, indicating genetic/chromosomal instability associated with high malignancy, before the development of cisplatin resistance. These results suggest that low doses of cisplatin administration in the clinical setting may promote malignancy and caution should be used with this type of treatment.

## 1. Introduction

Cisplatin is one of the most widely used cancer chemotherapy drugs. Cisplatin bonds covalently to the N7 positions of purine bases in DNA, creating intrastrand and interstrand cross-links that interfere with DNA replication and transcription, leading to cell cycle arrest and apoptosis [1–4]. The mechanism of action of cisplatin primarily involves the formation of DNA adducts which activate various cellular pathways, including the ATR (ataxia telangiectasia and Rad3-related) pathway, leading to DNA damage response and repair mechanisms [5]. Early effects of cisplatin include the formation of DNA cross-links and inhibition of DNA synthesis, while late effects involve the induction of apoptosis and cell death [6]. Cisplatin targets rapidly dividing cancer cells, but it also affects healthy cells, particularly those in the bone marrow, gastrointestinal tract, and renal tissues, leading to side effects such as nephrotoxicity, ototoxicity, and myelosuppression [7].

In clinical settings, low-dose cisplatin is often used in combination with other chemotherapeutic agents or radiation to enhance therapeutic efficacy while reducing overall toxicity [8]. This approach is particularly important for patients who are unable to tolerate high doses due to pre-existing conditions or severe side effects [1]. It is crucial to acknowledge that cisplatin also poses significant occupational hazards to healthcare workers who handle and administer the drug. Accidental exposure to cisplatin can result in contact dermatitis, reproductive toxicity, and has been associated with potential carcinogenicity among medical personnel [9]. These risks underscore the importance of adhering to strict safety protocols, including the use of personal protective equipment (PPE) and engineering controls, to minimize exposure and protect healthcare workers [10]. Examining the effects of low-dose cisplatin on DNA is crucial because even sub-toxic doses can induce significant cellular and molecular changes that may influence treatment outcomes [11]. Understanding these effects helps optimize dosing regimens and improve the therapeutic index of cisplatin, minimizing adverse effects while maximizing anticancer activity [12].

The effects of cisplatin on DNA, nuclei, nucleoli, and chromosomes have been studied *in vitro* by treating cells with increasing concentrations of cisplatin to induce cisplatin resistance. Thus far, the majority of published knowledge on cisplatin-caused DNA abnormalities has been based on cell lines characterized by acquired resistance [1,2,13]. These studies have demonstrated that cisplatin at high concentrations causes extensive nuclear, nucleolar, and chromosomal abnormalities [1,13–15].

Despite the widespread clinical use of low-dose cisplatin to minimize toxicity while maintaining efficacy, its effects on nuclei, nucleoli, and chromosomes remain underexplored. This study focuses on investigating these early effects at sub-toxic cisplatin concentrations *in vitro*. It was decided to use low cisplatin concentrations from 0.02 to 0.2 μg/ml on HeLa cells, while reported levels of the cisplatin IC50 usually range between 0.5 to 8 μg/ml (e.g. [1,16–19]).

This research aims to elucidate the mechanisms by which sub-toxic cisplatin levels induce genetic and chromosomal instability in HeLa cells, potentially contributing to increased malignancy prior to drug resistance development. Insights gained from this study may inform strategies for the safer and more effective use of low-dose cisplatin in clinical therapy.

## 2. Materials and methods

### 2.1. Cell lines

The HeLa cell line used in the present study was produced by single-cell cloning and maintained in McCoy’s 5a medium supplemented with 10% FCS, 10^5^ IU/l of penicillin, 10^5^ μg/l of streptomycin and 2 mg/l of amphotericin B. The cells were kept at 37°C in a humidified atmosphere containing 5% CO_2_. This cloned cell line had a replication time of 16 hours and viability of 97±1%. Cells were free of mycoplasma and cytomegalovirus. Viable cells were counted using the trypan-blue exclusion test. Replication time was determined as previously described [20]. The use of HeLa cells, a well-characterized human cervical cancer cell line, was due to their established responsiveness to cisplatin and their relevance in studying mechanisms of drug resistance and chromosomal instability.

### 2.2. Procedure for the development of cisplatin-sensitized cell lines

A total of 5×10^5^ cells attached to the surface of 5 ml plastic flasks were exposed to gradually increasing sub-toxic doses of cisplatin, starting at 0.01 μg/ml and increasing concentration every 7th day to 0.02, 0.03, 0.05, 0.07, 0.08, 0.1 (designated as HeLaC0.1), and 0.2 μg/ml (HeLaC0.2). HeLaC0.1 and HeLaC0.2 represented cell lines at very early stages of cisplatin resistance and were more accurately designated ‘cisplatin-sensitized’ cells. The above cisplatin concentrations were very low since viability for doses from 0.01 to 0.2 μg/ml was not lower than 88% compared to parental untreated HeLa cells. However, in the course of the study, it was soon noted that even at very low cisplatin concentrations, the cells reacted drastically, presenting changes in parameters associated with chromosomal instability. Cells sensitized to 0.02, 0.03, 0.05, 0.08, 0.1 and 0.2 μg/ml cisplatin were maintained at these concentrations in McCoy’s 5a medium for 7 days and then used in the described experiments. HeLa cells already sensitized for 7 days to low concentrations of cisplatin (0.02 or 0.08 μg/ml) were transferred to the next concentration level (0.03 or 0.1 μg/ml), respectively, for 24 hours and then transferred to cisplatin-free medium for 24 hours before processing for chromosomal analysis. This procedure enabled the study of the effects of 24-hour cisplatin treatment during all cell cycle phases for a period extended to three cell replication times (total 3 × 16 hours). The second period of 24-hour cisplatin-free culture facilitated the study of only cells with persisting viability after the first 1–1½ replications (first 24 hours) in cisplatin-containing medium. Cisplatin (Platinol) was purchased from Bristol-Myers Squibb Co., New York, NY, USA.

### 2.3. Techniques to observe choromosomal, nuclear and nucleolar abnormalities due to low-concentration of cisplatin

Chromosome preparation and stainings have been previously described [21]. Twenty-four hours before processing for chromosome preparation, cells were cultured in cisplatin-free medium. Thus, cells were cultured without cisplatin for approximately 1½ cell generation before subjecting them to colcemid shock. Cells were harvested and prepared for cytogenetic analysis at specific time points following cisplatin treatment. Chromosome preparations were made by standard procedures involving hypotonic treatment, fixation, and Giemsa staining for nuclear and chromosomal abnormalities [20,22,23]. The same slides stained with Giemsa were used for counting and examining nuclei, chromosomal number, chromosomal abnormalities, and chromosomal segregation. Nucleoli and nucleolar-organizing regions (NORs) were studied in silver-stained chromosomal preparations. Microscope slides were scanned and records of all cells and metaphases in consecutive fields were kept. Overlapping cells, or metaphases with difficulty in counting chromosomes were not counted. To ensure the reliability and reproducibility of the results, the procedure described in 2.2 was repeated using a separate initial HeLa sample under identical experimental conditions. Slides from each experiment were scored by three observers and any discrepancies in the scoring were resolved through joint review and discussion. A Zeiss microscope (Carl Zeiss, Jena, Germany) equipped with a digital camera was used for all observations.

### 2.4. Statistical analysis

Data are presented as counts and percentages. Categorical variables were compared among the groups using chi-square test or Fisher’s exact test. Post hoc Bonferroni correction was applied for pairwise comparisons. A-p value below 0.05 or 0.0125 with Bonferroni correction was regarded as statistically significant. Statistical analysis was performed using Stata 15.0 software (StataCorp LP,. College Station, TX, US).

## 3. Results

### 3.1. Low-dose cisplatin causes no difference in replication time and only a slight decrease in the viability of HeLa cells

The replication time of HeLa cells treated with subtoxic concentrations of cisplatin (0.01 to 0.2 μg/ml) did not alter. Viability was reduced by 6–8% in HeLaC0.1 and HeLaC0.2 cell lines and by 10–12% in the cells treated with a step increase in cisplatin concentration (0.02 to 0.03 and 0.08 to 0.1 μg/ml). These lower viabilities may be attributed to abnormal cell divisions leading to non-viable cells.

### 3.2. Abnormal nuclei in cisplatin-sensitized cells

**Fig 1:**
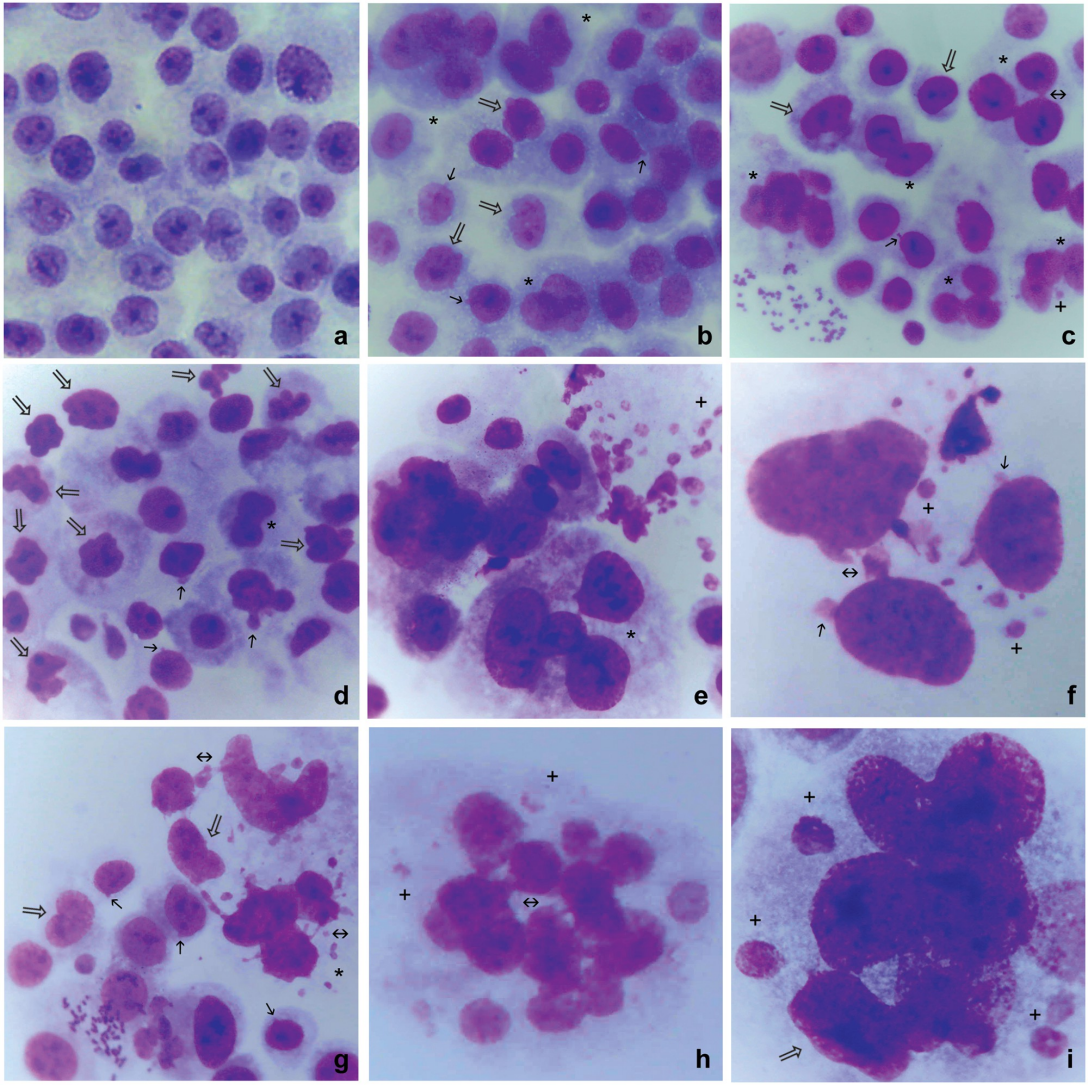
Morphological features of nuclei from untreated and low-concentration cisplatin-treated HeLa cells. a: Untreated HeLa parental cells. b: HeLaCO.1. c: HeLaCO.2. d: HeLa cells sensitized to 0.02 μg/ml and transferred to 0.03 μg/ml cisplatin. e: Multinucleated HeLa cells with abnormal and fragmented nuclei after transfer from 0.02 to 0.03 μg/ml cisplatin. f: Abnormal HeLa nuclei with blebs, chromatin bridges and micronuclei after transfer from 0.02 to 0.03 μg/ml cisplatin. g: Abnormal HeLa nuclei with blebs, chromatin bridges, and micronuclei after transfer from 0.08 to 0.1 μg/ml cisplatin. h: Multinucleated HeLaCO.1 cell with chromatin bridges, and micronuclei. i: Multinucleated HeLaCO.2 cells with abnormal nuclei and four large-size micronuclei. Giemsa; a, b, c, d, g, ×220; e, h ×420; f, i, ×1,000. Marks in the figures: ➝: blebs; ⇒: abnormal nuclear shape; ↔: chromatin bridge; *: multinucleated cell; +: micronucleus.

The parental untreated HeLa cell line exhibited 97.13% round or oval-shaped nuclei with one to three round nucleoli (Fig 1a). Abnormal nuclei (abnormal shape, blebs, fragmented or pulverized nuclear material, micronuclei, or multiple nuclei) were present in 2.86% of the untreated cells (Table 1).

**Table 1:** Morphology of nuclei in control untreated HeLa cells (0.0), HeLa cells sensitized to cisplatin at 0.1 μg/ml, 0.2 μg/ml (HeLaC0.1/HeLaC0.2) and HeLa cells sensitized for 7 days to 0.02 μg/ml (or 0.08 μg/ml), transferred to 0.03 μg/ml (or 0.1 μg/ml) cisplatin for 24 hours and then to cisplatin-free medium for 24 hours before chromosome preparation.

Cisplatin conc. (μg/ml)	Cells with normal nuclei (including large nuclei) ^a^	Cells with a single abnormal nucleus	Cells with one abnormal nucleus and 1–3 micronuclei	Binucleated	Trinucleated	Multinucleated	*p*-Values^#^
HeLa 0.0	97.13% (1490)	1.50% (23)	0.13% (2)	0.85% (13)	0.19% (3)	0.19% (3)	
HeLaC0.1	75.61% (871)	14.32% (165)	2.52% (29)	5.21% (60)	1.47% (17)	0.87% (10)	<0.001
HeLaC0.2	72.72% (965)	15.60% (207)	3.62% (48)	6.56% (87)	0.60% (8)	0.90% (12)	<0.001
0.02–0.03^b^	39.72% (1147)	47.89% (1383)	4.33% (125)	2.87% (83)	1.28% (37)	3.91% (113)	<0.001
0.08–0.1^b^	37.13% (1240)	55.70% (1860)	1.20% (40)	2.69% (90)	0.72% (24)	2.57% (86)	<0.001

Normal nuclei were considered those with round or oval shape. Abnormal nuclei were considered those with altered shape, blebs, and fragmented or pulverized nuclear material. Cells with one abnormal nucleus and 1–3 micronuclei were counted separately. The number of cells is given in parentheses.

^#^: p-Values from pairwise comparisons to the untreated cells.

^a^: Including cells with both abnormal and normal nuclei.

^b^: Cisplatin concentration in HeLa cells sensitized to 0.02 μg/ml (or 0.08 μg/ml) was increased to 0.03 μg/ml (or 0.1 μg/ml) and cells were examined as described in the Materials and Methods.

Both cisplatin-sensitized cell lines, HeLaC0.1 and HeLaC0.2, exhibited a prominent decrease of normal nuclei and an increase of all nuclear abnormalities, compared to parental HeLa, as shown in Table 1.

Notably, the increase of nuclear abnormalities and multinucleated cells after increasing the cisplatin concentration from 0.02 to 0.03 or from 0.08 to 0.1 μg/ml (“sudden challenge”) was even more prominent compared to HeLaC0.1 and HeLaC0.2 cells (Table 1). These findings indicate that a sudden increase in the cisplatin level causes an immediate cellular response within the next 2–3 cell generations, involving extensive nuclear abnormalities. Fig 1b–1i depicts nuclear abnormalities representative of those found after exposure to various levels of cisplatin.

## 3.3. Increase of chromosome number in cisplatin-sensitized cells (Table 2)

**Table 2:** Distribution of chromosome numbers in HeLa cells sensitized to sub-toxic concentrations of cisplatin.

Cisplatin conc. (μg/ml)	% of metaphases					*p*-Values^#^
	Chromosome numbers^a^					
	≤60	61–80	81–100	101–140	>140	
HeLa 0.0	1.59% (7)	91.34% (401)	3.19% (14)	3.42% (15)	0.45% (2)	
HeLaC0.1	0.97% (4)	65.53% (270)	29.37% (121)	3.16% (13)	0.97% (4)	<0.001
HeLaC0.2	2.31% (10)	69.75% (302)	19.17% (83)	4.85% (21)	3.93% (17)	<0.001
0.02–0.03^b^	3.38% (11)	68.92% (224)	7.69% (25)	17.23% (56)	2.77% (9)	<0.001
0.08–0.1^b^	2.59% (9)	81.27% (282)	8.07% (28)	6.05% (21)	2.02% (7)	<0.001

^#^: p-Values from pairwise comparisons to the untreated cells.

^a^: 382±157 metaphases were examined in each case. Because of frequent chromosome overlapping and segregation, the chromosome number was categorized into five groups.

^b^HeLa cells sensitized to 0.02 μg/ml or 0.08 μg/ml cisplatin were then exposed to cisplatin at 0.03 μg/ml or 0.1 μg/ml, respectively, and metaphases were prepared as described in the Materials and Methods.

In comparison with the control untreated HeLa cells, all cisplatin-sensitized cells showed a decrease in the proportion of cells with 61–80 chromosomes and an increase of cells with 81–100, 101–140 and >140 chromosomes. These counts indicate an obvious tendency for an increase in polyploid cells as an immediate response to low cisplatin concentrations.

### 3.4. Abnormal chromosome segregation in cisplatin-sensitized cells (Table 3; Fig 2a–2c)

**Fig 2:**
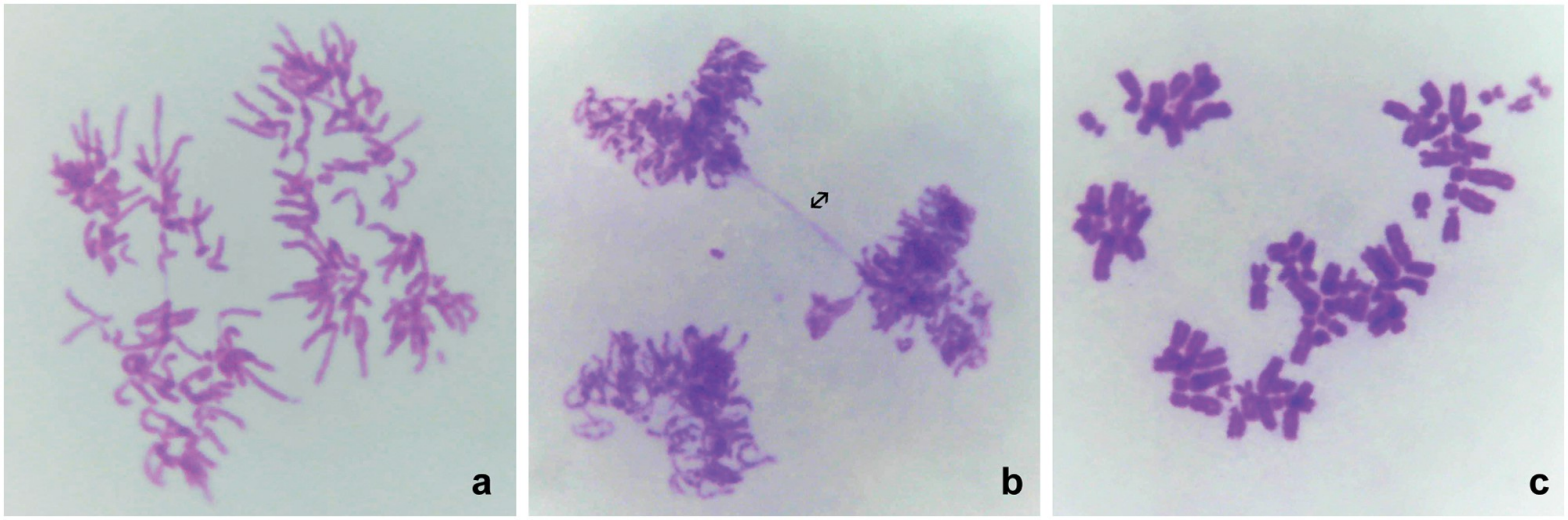
Abnormal chromosomal segregation. a: HeLaC0.1, chromosomal segregation following tetrapolar mitosis. b: HeLaC0.1, abnormal tripolar mitosis with anaphase bridge (↔). c: HeLaC0.2, segregation of metaphase chromosomes.

**Table 3:** Percentage of metaphases displaying abnormally segregated chromosomes in HeLa cells sensitized to 0.1 μg/ml and 0.2 μg/ml cisplatin and control HeLa cells. 400±50 metaphases were counted.

Cisplatin concentration (μg/ml)	% of metaphases with segregated chromosomes	*p*-Values^#^
HeLa 0.0	8.2%	
HeLaC0.1	46.6%	<0.001
HeLaC0.2	67.5%	<0.001

^#^: p-Values from pairwise comparisons to the untreated cells.

A high frequency of abnormal chromosomal segregation in metaphases was observed in HeLaC0.1 cells (46.6%) and even higher in HeLaC0.2 cells (67.5%) (Table 3). Anaphase bridges, tripolar and tetrapolar mitoses were also very frequent (Fig 2a and 2b).

### 3.5. Abnormal morphology and increased number of nucleoli in cisplatin-sensitized cells

**Fig 3:**
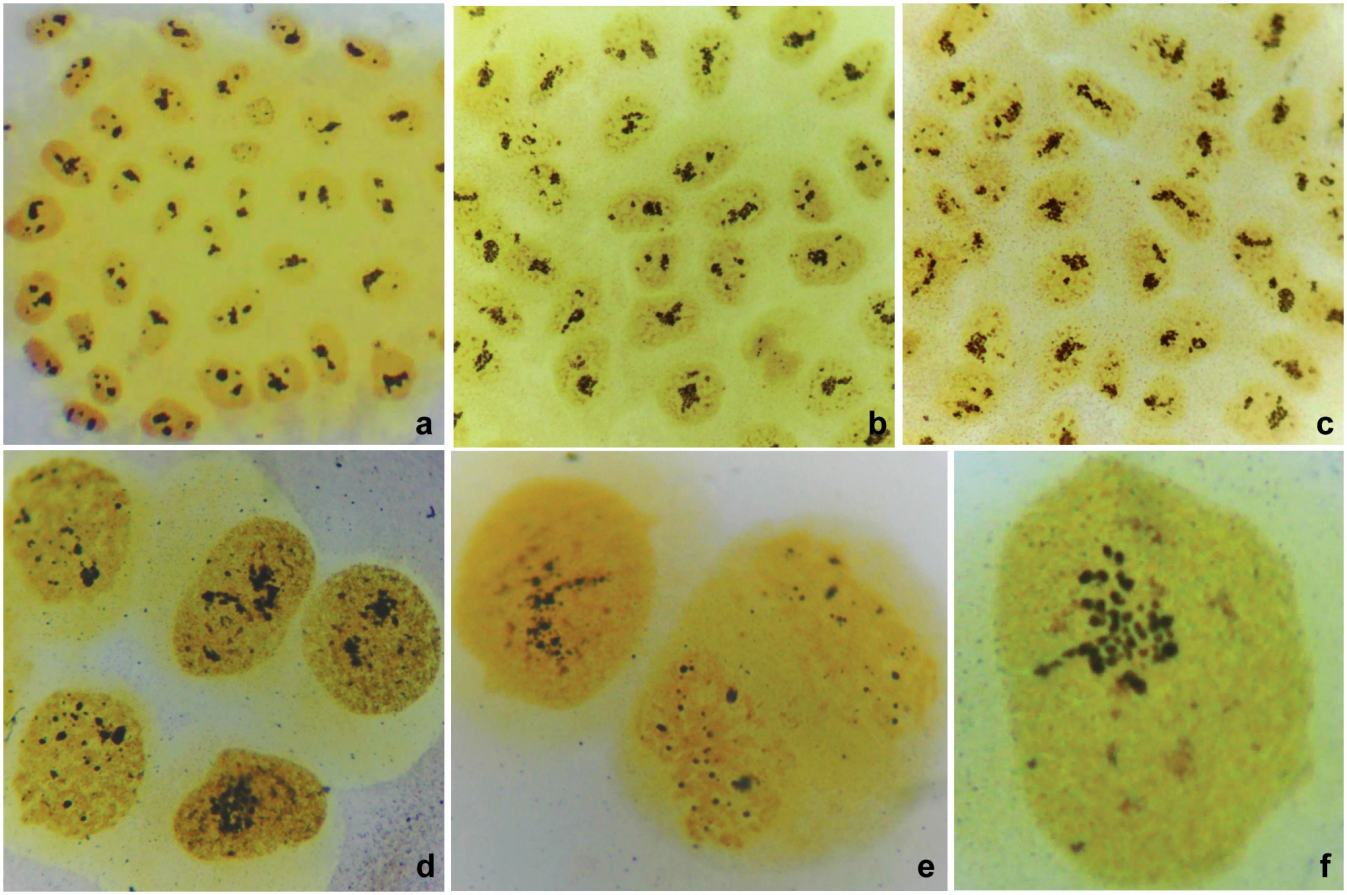
Morphological features of nucleoli. a: HeLa cells. b: HeLaC0.1 cells. c: HeLaC0.2 cells. d: Nucleoli in HeLa cells sensitized to 0.02 and transferred to 0.03 μg/ml cisplatin. Over 20 dots of nucleolar organizing regions (NORs) can be seen in the nucleus at the lower left corner. e: HeLaC0.2 cells. Left, a nucleus with over 20 NOR dots. Right, late anaphase with over 20 NOR dots in the newly formed nuclei. f: Nucleus of a HeLa cell sensitized to 0.08 and transferred to 0.1 μg/ml cisplatin. Over 25 nucleoli are visible with variable sizes, appearing as single dots or assemblies of 2–3 dots. Silver staining; a, ×100; b, ×110; c, ×110; d, ×500; e, ×700; f, ×1,200.

The interphase nuclei of parental untreated HeLa cells exhibited 1–3 large, round nucleoli, rarely accompanied by 1–3 small nucleoli (Figs 1a and 3a), all darkly stained with Giemsa or silver staining.

Fig 3 depicts nucleolar alterations in shape, morphology, and number in cisplatin-sensitized HeLa cells.

HeLaC0.1 cells exhibited one large, irregularly-shaped and several (n = 2–7) small nucleoli (Fig 3b). HeLaC0.2 cells exhibited 1–3 large, irregularly-shaped nucleoli and many (n = 3–12) small nucleoli (Fig 3c). In both these cell lines, the irregularly-shaped nucleoli showed a peculiar morphology, giving the impression of an aggregate of many smaller nucleoli resembling a “bunch of grapes”. Small nucleoli were of variable size (Fig 3).

When HeLa cells, already sensitized to 0.02 μg/ml (or 0.08 μg/ml) cisplatin, were treated with 0.03 μg/ml (or 0.1 μg/ml), many more (often over 20) small nucleoli, clearly separated from each other, were observed, giving the impression that the large irregular nucleoli were further disaggregated (Fig 3d and 3f).

### 3.6. Increased NORs on chromosomes of cisplatin-sensitized cells

**Fig 4:**
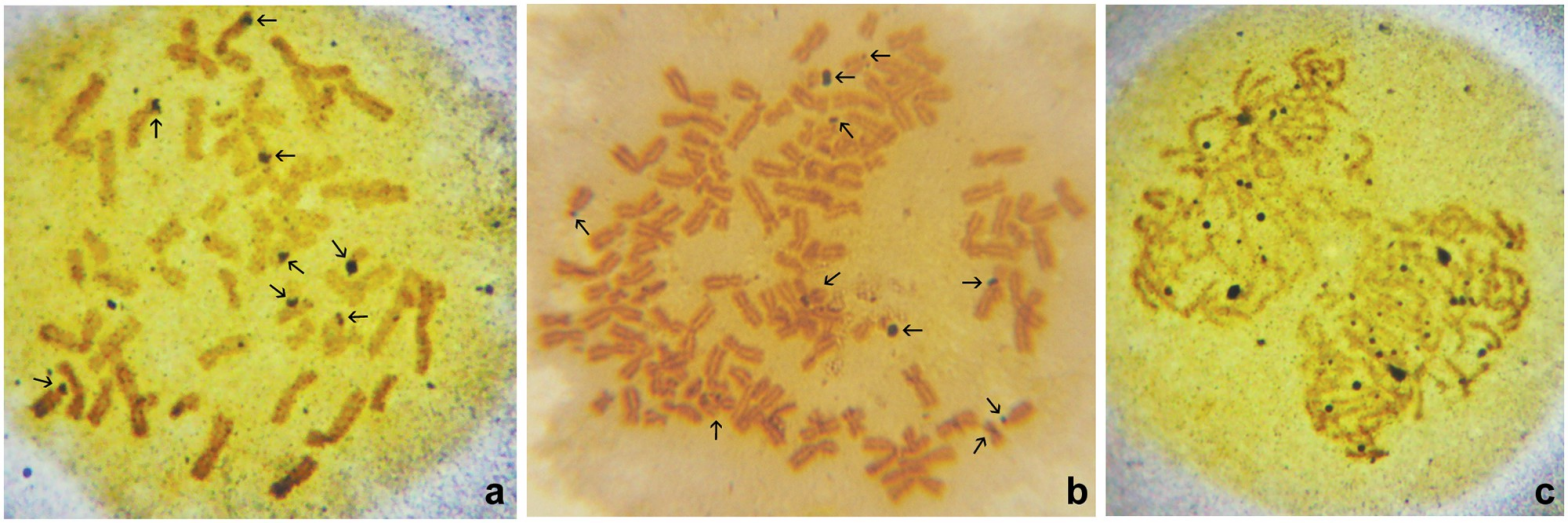
Nucleolar organizing regions (NORs) on chromosomes. a: Metaphase of a HeLa cell sensitized to 0.02 and transferred to 0.03 μg/ml cisplatin. Distinctly activated NORs are present on eight acrocentric chromosomes (arrows), with one of them being the most intensely stained on a small acrocentric chromosome. b: Metaphase of a HeLaC0.2 cell showing 10 NORs (arrows), with two of them intensely stained on two small acrocentric chromosomes. c: Early anaphase of a HeLa cell sensitized to 0.02 and transferred to 0.03 μg/ml cisplatin. Over 24 NORs of various sizes are visible, with two of them being more intensely stained.

NORs can be located on small acrocentric chromosomes stained with Giemsa as diffused light-staining at the ends of short chromosome arms. However, NOR staining is more intense with silver staining in most acrocentric chromosomes. In untreated parental HeLa cells, NORs were usually stained on 3–7 chromosomes, while in HeLaC0.1 and HeLaC0.2, and cells 48 hours after a sudden cisplatin challenge (0.02 to 0.03 or 0.08 to 0.1 μg/ml), stained NORs increased to 4–12 (Fig 4a and 4b). The increase of NORs in metaphases of cisplatin-sensitized cells correlated with the disaggregation of the large irregular nucleoli into smaller ones in interphase cells (Figs 3d–3f, 4a and 4b). One or two small acrocentric chromosomes were always the carriers of a larger NOR in all the metaphases or anaphases of the cisplatin-sensitized cells examined (e.g. Fig 4a and 4b). An even higher number of intensively stained NORs was observed during anaphase (Figs 3e and 4c).

### 3.7. Extensive multiple-type chromosomal abnormalities in cisplatin-sensitized cells (Table 4)

**Fig 5:**
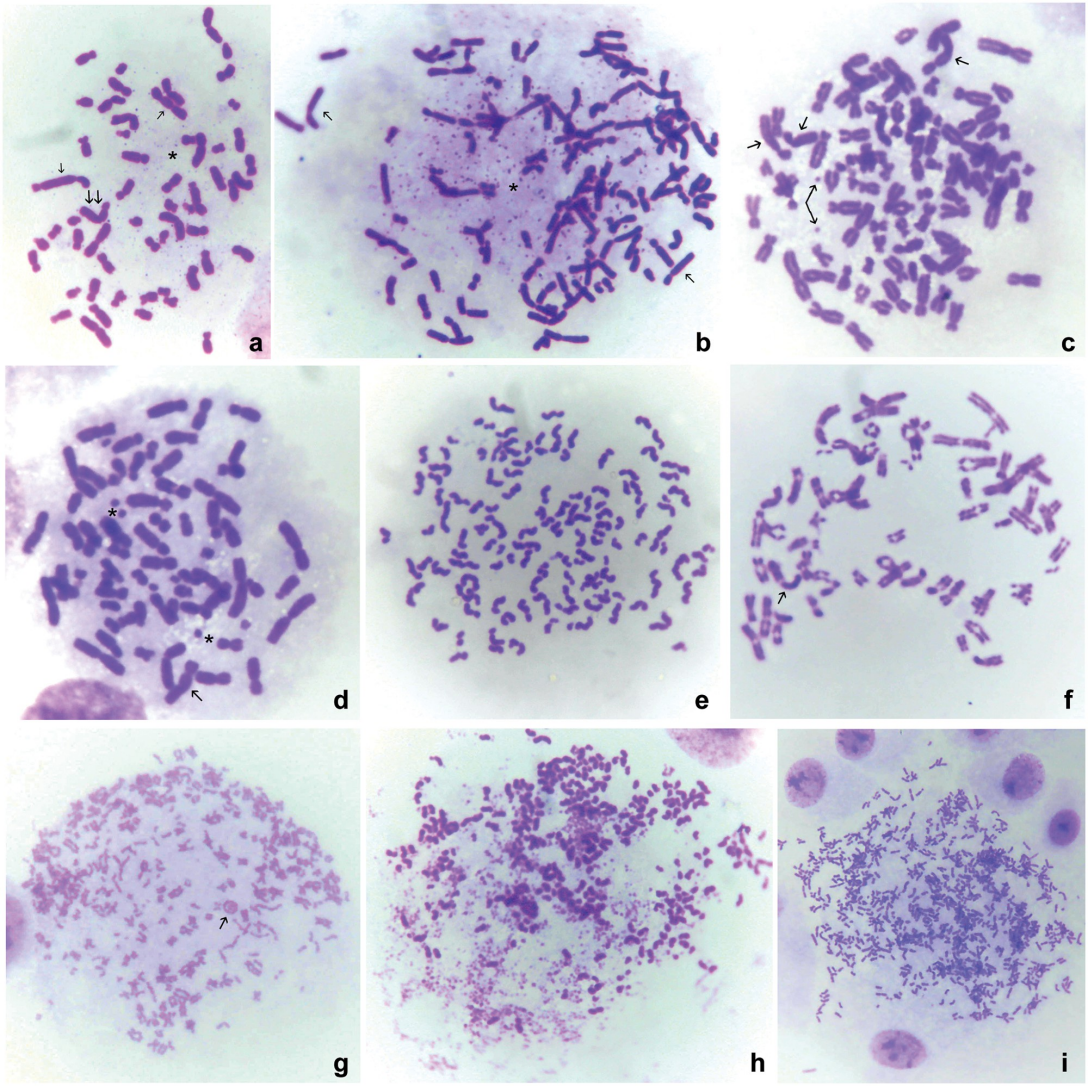
Chromosomal abnormalities. a: Metaphase of a cell sensitized to 0.02 and transferred to 0.03 μg/ml cisplatin, containing two dicentric chromosomes (single arrows), one marker (double arrow), and numerous double minutes (DMs) (asterisk). b: Metaphase of a cell sensitized to 0.08 and transferred to 0.1 μg/ml cisplatin, containing numerous DMs, two marker chromosomes (arrows) and chromosome segregations. c: Metaphase of a HeLa cell sensitized to 0.02 μg/ml cisplatin, containing two marker chromosomes (short arrows) and one DM with two far-separated dots (arrows). d: Metaphase of a HeLa cell sensitized to 0.02 μg/ml cisplatin, containing three large DMs (asterisks) and one marker chromosome (arrow). e: Metaphase of a HeLa cell sensitized to 0.08 μg/ml cisplatin, containing sister chromatid separations. f: Metaphase of a HeLa cell sensitized to 0.08 μg/ml cisplatin, with two DMs (long arrows), one marker chromosome (short arrow), and several chromosomes with a centromere separation. g: Metaphase of a HeLa cell sensitized to 0.07 μg/ml cisplatin, showing a ring chromosome (arrow), sister-chromatid separations, and unaffected chromosomes. h: Metaphase of a HeLa cell sensitized to 0.02 μg/ml cisplatin, showing sister-chromatid separations, chromosome pulverization, and chromosome segregations. i: Metaphase of a HeLa cell sensitized to 0.02 μg/ml cisplatin, showing over 600 chromosomes and chromosomal segregations.

**Fig 6:**
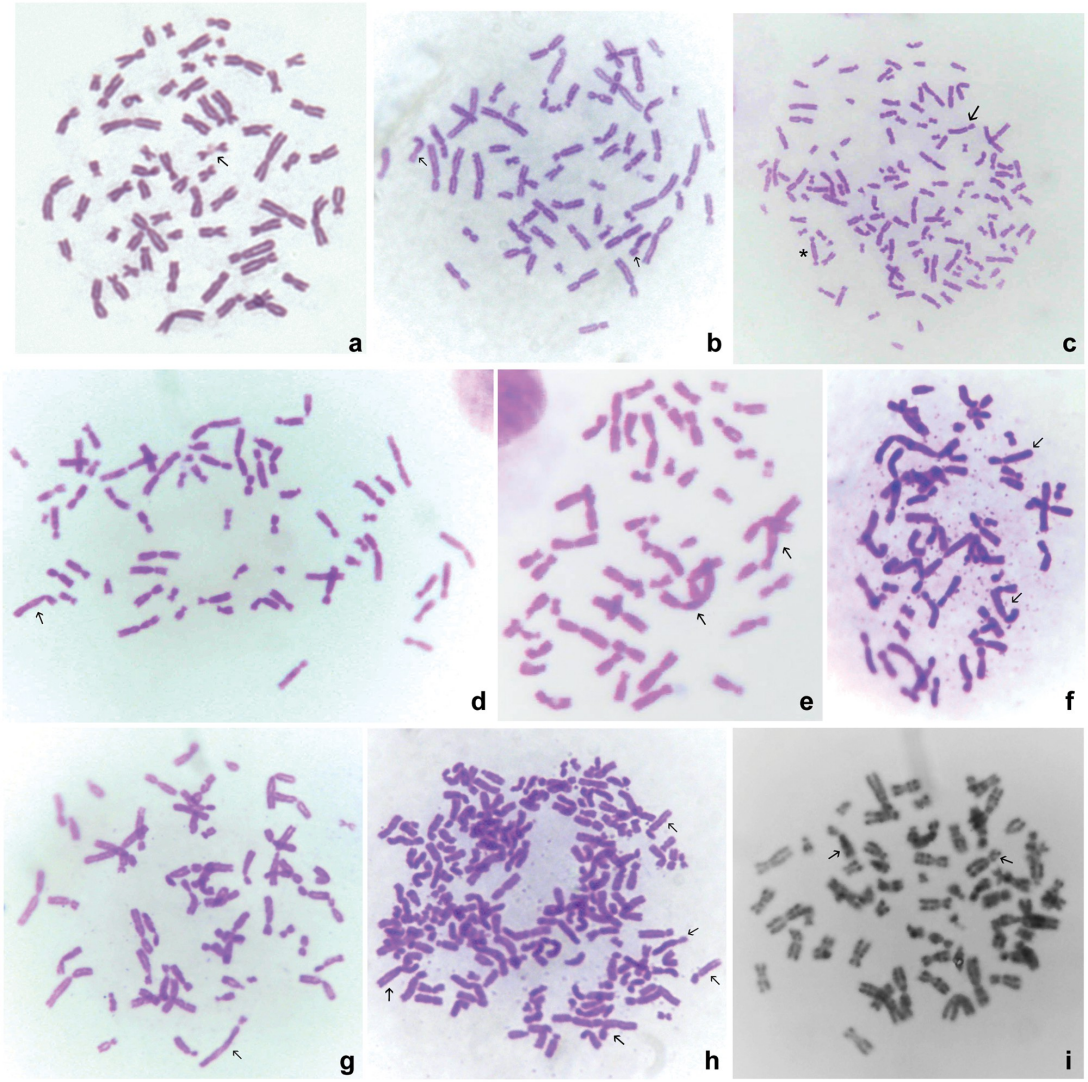
Metaphases with marker chromosomes. a: HeLa cell (control) metaphase, with two associated small acrocentric chromosomes (arrow). b: HeLaC0.2 cell metaphase containing two marker chromosomes (arrows). c: HeLaC0.2 cell metaphase containing one dicentric (asterisk) and one marker chromosome (arrow). d: Metaphase of a HeLa cell sensitized to 0.02 μg/ml cisplatin, containing one marker chromosome (arrow). e: Metaphase of a HeLa cell sensitized to 0.02 and transferred to 0.03 μg/ml cisplatin, containing two marker chromosomes (arrows). f: Metaphase of a HeLa cell sensitized to 0.08 μg/ml and transferred to 0.1 μg/ml cisplatin, containing numerous double minutes (DMs) and two marker chromosomes (arrows). g: Metaphase of a HeLa cell sensitized to 0.08 μg/ml and transferred to 0.1 μg/ml cisplatin, containing one marker chromosome with extended long arm (arrow). h: Metaphase of a HeLa cell sensitized to 0.02 μg/ml cisplatin, with over 140 chromosomes including DMs and more than four marker chromosomes (arrows). i: G-banding of metaphase of a HeLa cell sensitized to 0.08 μg/ml cisplatin, showing two marker chromosomes (arrows).

The parental untreated HeLa cells exhibited 5.71% of metaphases with fragmentation-induced abnormalities. A prominent increase of metaphases with double minutes (DMs), chromatid exchanges, and chromatid separations, was observed in both HeLaC0.1 and HeLaC0.2 cells, though this increase was not statistically significant. Nevertheless, the percentage of cells with chromosomal abnormalities was significantly elevated in all experiments of transfer from a lower to a higher cisplatin concentration (0.02 to 0.03 or 0.08 to 0.1 μg/ml), compared to the untreated cells (Table 4; Figs 5 and 6). DMs were present in variable numbers and sizes (Figs 5a–5d, 5f, 6f and 6h) and with variable distances between the two separated dots (Figs 5c, 6f and 6h). Pulverization of chromosomes was observed only in polyploid HeLaC0.2 metaphases (Fig 5h). Dicentrics and chromosome pulverization were observed in HeLaC0.2 and all experiments of transfer from a lower to a higher concentration of cisplatin (Table 4, Figs 5a and 6c).

**Table 4:** Percentage of cisplatin-sensitized HeLa cells with structural chromosomal abnormalities in metaphase.

Cisplatin conc. (μg/ml)	Metaphases examined, n	DMs	Dots	Dicentrics	Breaks and gaps	Chromatid exchanges, chromatid separations, and rings	Distorted or fragmented chromosomes	Pulverized chromosomes^a^	Total cells with chromosomal abnormalities	*p*-Values^#^
HeLa 0.0	35	0	0	0	2.86 (1)^b^	0	2.86 (1)	0	5.71% (2)	
HeLaC0.1	52	9.62 (5)	1.92 (1)	0	1.92% (1)	5.77% (3)	0	0	19.23% (10)	0.073
HeLaC0.2	50	16.00% (8)	0	4.00% (2)	0	4.00% (2)	0	2.00% (1)	26.00% (13)	0.016
0.02–0.03^c^	56	16.07% (9)	3.57% (2)	3.57% (2)	12.50% (7)	17.86% (10)	3.57% (2)	5.36% (3)	62.49% (35)	<0.001
0.08–0.1^c^	56	21.43% (12)	1.79% (1)	5.36% (3)	10.71% (6)	19.64% (11)	3.57% (2)	7.14% (4)	69.64% (39)	<0.001

DMs: Double minutes.

^#^: p-Values from pairwise comparisons to the untreated cells.

^a^In polyploid metaphases.

^b^Number of metaphases is given in parentheses.

^c^HeLa cells sensitized to 0.02 μg/ml or 0.08 μg/ml cisplatin were then exposed to cisplatin at 0.03 μg/ml or 0.1 μg/ml, respectively, and metaphases were prepared as described in the Materials and Methods.

A unique submetacentric marker chromosome was observed in numbers ranging from 1 to ≥4 in the majority of the cells sensitized to cisplatin (Table 5). This marker was recognized and characterized by: (a) a bent short arm, resembling a walking stick (Figs 5b, 5c, 5f, 6b, 6d, 6f and 6h); (b) an arc-shaped long arm (Figs 5a–5d, 6b, 6c, 6e and 6h); (c) twisting of the two chromatids in the middle of the long arm, and (d) a rarely- seen elongation of the long arm (Fig 6g). Disfigurements were noticed in both arms of the marker, and in G-banding, the long arm appeared darkly stained (Fig 6i). The marker did not appear in parental HeLa cells but appeared in varying numbers in both HeLaC0.1 and HeLaC0.2 metaphases (Figs 5a–5d and 6). One or three markers were rarely seen, while two markers were seen in a high percentage of both HeLaC0.1 and HeLaC0.2 cells (40.38% and 36.00%, respectively). When the cisplatin concentration was increased as a sudden challenge from 0.02 (or 0.08) to 0.03 (or 0.1) μg/ml, a high percentage of metaphases with two markers (44.64% and 46.43%, respectively), was also seen, while metaphases with one or three markers were very rare. Four or more markers were found in all polyploid cells.

**Table 5:** Percentage of metaphases of HeLa cells in very early stages of sensitization to cisplatin-containing one or more submetacentric marker chromosomes characterized by a bent short arm and frequent twisting of the two chromatids in the middle part of the long arm.

Cisplatin conc. (μg/ml)	Metaphases examined, n	Number of submetacentric marker chromosomes				Total metaphases containing marker chromosomes	*p*-Values^#^
		1	2	3	≥4^a^		
HeLa 0.0	35	0	0	0	0		
HeLaC0.1	52	1.92% (1)^c^	40.38% (21)	3.85% (2)	7.69% (4)	53.84% (28)	<0.001
HeLaC0.2	50	2.00% (1)	36.00% (18)	4.00% (2)	4.00% (2)	46.00% (23)	<0.001
0.02–0.03^b^	56	0	44.64% (25)	1.79% (1)	16.07% (9)	62.50% (35)	<0.001
0.08–0.1^b^	56	3.57% (2)	46.43% (26)	1.79% (1)	14.29% (8)	66.08% (37)	<0.001

^#^: p-Values from pairwise comparisons to the untreated cells.

^a^In polyploid metaphases.

^b^HeLa cells sensitized to 0.02 μg/ml or 0.08 μg/ml cisplatin were then exposed to cisplatin at 0.03 μg/ml or 0.1 μg/ml, respectively, and metaphases were prepared as described in the Materials and Methods.

^c^Number of metaphases is given in parentheses.

## 4. Discussion

Analysis of genomic/chromosomal instability in cancer itself and in cisplatin-resistant human cancer cell lines has implicated almost all chromosomes in the relevant mechanisms of development of drug resistance, either as specific chromosome abnormalities or as a result of a generalized effect on the whole genome [24–28]. This aspect has been supported by several investigations implying that polyploid cells may generate aneuploid drug-resistant [29–34] and potentially metastatic progeny [29,32–34], a heterogeneous cell population characterized by extensive genomic reorganization [26]. Therefore, cancer cells seem to possess an inherent ability to develop–within their population–polyploid or multinucleated cells, especially under diverse conditions that threaten their survival [31,35]. These conditions include the culture of cancer cells with cytotoxic anticancer agents, such as methotrexate [30] or cisplatin [36], and ionizing radiation [37], as well as when co-cultured with proliferating fibroblasts [38,39]. Polyploid cells have also been described in biopsies of many tumor types [32,40].

The present results show that HeLa cells growing continuously at low sub-toxic concentrations of cisplatin (HeLaC0.1 and HeLaC0.2) acquired a karyotype characterized by a decrease in levels of aneuploidy (chromosomes 61–80) and an increase in cells with high levels of aneuploidy and polyploidy (containing 81 to over 140 chromosomes) (Table 2). Additionally, an increase in binucleated, trinucleated, and multinucleated cells was concurrently observed, and, remarkably, even greater increases were observed following a stepping up of the cisplatin concentration (from 0.02 to 0.03 and from 0.08 to 0.1 μg/ml) (Table 1). Both these observations in the number of chromosomes and nuclei highlight the induction of polyploidization as an early phenomenon within the response of HeLa cells to sub-toxic concentrations of a DNA-damaging agent, cisplatin. This phenomenon also coincided with the profound differences in NORs and nucleoli (Figs 3 and 4), increases in metaphases with abnormally-segregated chromosomes (Table 3; Fig 2), and increases in the frequency of all types of chromosomal abnormalities (Table 4; Fig 5), which have been repeatedly associated with drug resistance, DMs [30,41–43], and dicentrics [30,44]. DMs, consisting of a form of extra-chromosomal DNA, have been shown to originate from homogeneously-stained chromosome regions by chromothripsis [25,27,42], to contain amplified genes, and to support cell growth under unfavorable or survival-threatening conditions [43,45,46].

The nuclear abnormalities induced by sub-toxic cisplatin concentrations were remarkable in all the experiments of the present work (Table 1, Fig 1b–1i). However, since only blebs extruding from the nuclear periphery were microscopically obvious (e.g., Fig 1b and 1c, arrows) and able to be counted, the actual percentage of cells with single abnormal nuclei should be considered to be higher in all observations cited in Table 1. Nuclear blebbing is an important element in the evolution of new phenotypes [47] through the induction of nuclear fragility and instability [48].

Nucleolar morphology and NORs in metaphases were found to change drastically in cisplatin-sensitized HeLa cells showing intense silver staining (Figs 3 and 4), indicative of overproduction of rRNA. Similar manifestations of nucleolar overactivity and rRNA overproduction have been described in cisplatin-treated [49], cisplatin-resistant [50], and methotrexate-resistant [21] cells.

The association of all NOR-carrying chromosomes with nucleoli within interphase nuclei [51] was intensely disrupted in cisplatin-sensitized HeLa cells at all concentration levels (Figs 3d–3f, 4a and 4b), indicating that cisplatin also influences chromosomal positioning in the nucleus. Remarkably, the prominent silver staining on the two small acrocentric chromosomes in metaphase was correlated with two equally prominent nucleolar dots in daughter cells in anaphase (Figs 3e and 4c). Furthermore, anaphases of cisplatin-sensitized cells exhibited an even higher number of NORs (over 25) (Figs 3e and 4c), indicating an enhanced production of rRNA and activation of nucleolar genes.

The frequent observation of abnormal sister chromatid separation, involving centromere abnormalities (Fig 5e–5h), also implicates the harmful effects of cisplatin on the structure of the centromere, obviously through damage induced in rDNA. Disruption of centromere structure has been associated with genomic instability [52], and with irregular chromosomal movements during normal cell division [53].

Chromosome abnormalities of all types were observed in a profoundly higher percentage of metaphase cells, following a “sudden” or step increase of cisplatin concentration (0.02 to 0.03 and 0.08 to 0.1 μg/ml) (Fig 5a and 5b). Fig 5 shows representative metaphases containing chromosomal abnormalities, and Figs 5b, 5g–5I and 6h show representative polyploid metaphases of HeLa cells sensitized to a low concentration of cisplatin.

Finally, a unique morphologically-distorted chromosome was present and regularly replicated in a high percentage of the cisplatin-sensitized HeLa cells (Table 5; Figs 5a–5d and 6).

The present detailed examination of the nuclei, nucleoli, chromosome number, and chromosomal abnormalities at sub-toxic cisplatin concentrations suggests that cancer cells develop a response towards maintaining survival much earlier than the stage of acquired resistance. This response seems (a) to be an immediate reaction or an inherent trait of the cancer cells and (b) to bring cancer cells to a stage of genetic/chromosomal instability that will eventually result in the establishment of a cell population with a higher resistance level, and even more importantly possible greater malignancy, since increases in chromosomal abnormalities and chromosome instability are indicative of an increase in malignancy [31,32,34,35].

It should be noted that during cisplatin treatment, the concentration of cisplatin within a tumor may vary due to microenvironmental conditions (necrotic areas, insufficient angiogenesis system), differential absorption by cancer cells in different cell cycle phases, or due to cisplatin metabolism.

This work is important because it highlights the potential risks associated with low-dose cisplatin therapy, a common clinical practice aimed at reducing toxicity while maintaining efficacy. By revealing the early nuclear, nucleolar and chromosomal changes induced by sub-toxic cisplatin concentrations, this study provides crucial insights into the mechanisms that may lead to increased malignancy and resistance. Therefore, caution should be applied to long-term fluctuating doses of cisplatin treatment in the clinic that may lead to increased malignancy in tumors, quite early in treatment and long before resistance appears.

## 5. Conclusion

The present study demonstrates that sub-toxic concentrations of cisplatin induce extensive chromosomal, nuclear, and nucleolar abnormalities in HeLa cells. Abnormalities found in this research are associated with high malignancy and genetic instability, suggesting that even low doses of cisplatin can promote significant cellular changes before the development of resistance. These findings underscore the need for clinical caution when using long-term or fluctuating low-dose cisplatin regimens, as they may enhance tumor malignancy and complicate treatment outcomes. Further research is needed to explore the long-term effects of low-dose cisplatin and develop strategies to mitigate its potential risks.

## Abbreviations

NORs: nucleolar organizing regions
